# Discriminating patients with early-stage breast cancer from benign lesions by detection of oxidative DNA damage biomarker in urine

**DOI:** 10.18632/oncotarget.17831

**Published:** 2017-05-12

**Authors:** Cheng Guo, Xiaofen Li, Minfeng Ye, Fei Xu, Jiekai Yu, Cong Xie, Xiaoji Cao, Mengzhe Guo, Ying Yuan, Shu Zheng

**Affiliations:** ^1^ Cancer Institute (Key Laboratory of Cancer Prevention and Intervention, China National Ministry of Education), The Second Affiliated Hospital, Zhejiang University School of Medicine, Hangzhou, Zhejiang 310009, China; ^2^ Department of Gastrointestinal Surgery, Shaoxing People's Hospital, Shaoxing Hospital of Zhejiang University, Shaoxing, Zhejiang 312000, China; ^3^ College of Chemical Engineering, Zhejiang University of Technology, Hangzhou, Zhejiang 310014, China; ^4^ Jiangsu Key Laboratory of New Drug Research and Clinical Pharmacy, Xuzhou Medical College, Xuzhou, Jiangsu 221004, China; ^5^ Department of Medical Oncology, The Second Affiliated Hospital, Zhejiang University School of Medicine, Hangzhou, Zhejiang 310009, China

**Keywords:** breast cancer, benign lesion, biomarker, urine, mass spectrometry

## Abstract

Breast cancer is one of the most commonly diagnosed and death-related cancers in women worldwide. Mammography is routinely used for screening and invasive examinations such as painful tissue biopsies were recommended for patients with abnormal screening outcomes. However, a considerable proportion of these cases turn out to be benign lesions. Thus, novel non-invasive approach for discriminating breast cancer from benign lesions is desirable. Herein, we applied a high-throughput ultra performance liquid chromatography-electrospray ionization tandem mass spectrometry (UPLC-ESI-MS/MS) analysis to determine the oxidative DNA damage biomarker, 8-oxo-7,8-dihydro-2′-deoxyguanosine (8-oxodG) in urine samples from 60 patients with early-stage breast cancer (stage I, II), 51 patients with benign breast diseases and 73 healthy volunteers. We demonstrated that the concentration of urinary 8-oxodG in patients with early-stage breast cancer was significantly higher not only than that in healthy controls, but also than that in patients with benign breast diseases, whereas no significant difference of urinary 8-oxodG level was observed between benign breast diseases group and healthy control group. Moreover, there was significant difference between early-stage breast cancer group and non-cancerous group which consisted of benign breast diseases patients and healthy controls. Besides, logistic regression analysis and receiver operator characteristic (ROC) curve analysis were also performed. Our findings indicate that the marked increase of 8-oxodG in urine may serve as a potential biomarker for the risk estimation, early screening and detection of breast cancer, particularly for discriminating early-stage breast cancer from benign lesions.

## INTRODUCTION

Breast cancer is the one of most prevalent malignant disease in the world and, by far, the most frequent cancer among women both in the United States and China [1, 2]. It is also one of the most aggressive malignant tumors with high mortality. Early detection of breast cancer is a vital aspect of treatment because early stages (I, II) are easier to cure than later stages (III, IV). Currently, mammography is the most widely used approach for the scanning of breast cancer. Women with abnormal screening outcomes undergo further magnetic resonance imaging and tissue biopsies such as fine-needle aspiration, causing additional mental stress and costs. However, few percent of these women will have cancer whereas most cases turn out to be benign diseases. To avoid unnecessary expensive and invasive screening for those benign patients, a robust, accurate and non-invasive detection methodology for breast cancer, particularly for discrimination early cancer from benign lesions, is urgently needed.

Due to its great advantages in specificity, repeatability, sensitivity and accuracy, mass spectrometry-based analytical techniques play important roles in the field of biomarker discovery and have attracted great attention [3–7]. In the past decades, continuous efforts have been devoted into discovering novel biomarker for detection of breast cancer by liquid chromatography-electrospray ionization tandem mass spectrometry (LC-ESI-MS/MS) [8–12]. Recently, plasma lipidomics profiling revealed lipids biomarkers for distinguishing breast cancer from benign lesions [13, 14]. However, little attention has been paid to discriminating breast cancer from benign lesions by urinary biomarker although urinary metallomics analysis by inductively coupled plasma mass spectrometry (ICP-MS) has been reported [15].

Reactive oxygen species (ROS) are produced by endogenous oxygen metabolism, as well as after exposure to harmful environmental factors, such as ionizing radiation and chemical carcinogens [16]. Hydroxyl radical, hydrogen peroxide, superoxide anion and singlet oxygen are the most well-known ROS in biological systems. An imbalance between the production and scavenging of ROS, known as oxidative stress, will result in the damage of all macromolecules, including proteins, lipids and nucleic acids [17, 18]. Among these cellular biomolecules that may be attacked and modified by ROS, DNA has attracted great attention [19, 20]. It has been recognized that oxidative DNA damage can lead to cytotoxic effects and is implicated in aging and the pathogenesis of a variety of diseases such as neurodegenerative diseases, chronic inflammatory diseases, cardiovascular diseases, diabetes and cancer [21–23].

Increased ROS can cause oxidative base modifications in DNA and more than 20 different types of base lesions products have been identified [24]. Since guanine exhibits the lowest oxidation potential, the guanine residues of nucleic acid are more vulnerable to free radical, leading to the formation of 8-oxo-7,8-dihydro-2′-deoxyguanosine (8-oxodG) which has been commonly chosen as a biomarker of oxidative damage to DNA. The 8-oxodG residues in DNA can pair with adenine as well as cytosine during DNA replication, thus causing transversion-type mutations (GC to TA) [25]. This mutation constitutes the second most common somatic mutations found in human cancers. Therefore, the presence of 8-oxodG in cells may lead to mutagenesis and may thus be the major contributor to carcinogenesis.

In fact, persistent oxidative stress exists in cancer [26]. In the past few decades, 8-oxodG has been vastly investigated in tissues and cells in humans and animal models [27–30]. In previous studies, elevated levels of 8-oxodG were observed in cancer cell lines or cancerous tissues compared to normal cell lines or adjacent normal tissues. For example, the 8-oxodG levels in DNA isolated from breast cancer tissues were significantly higher than those from corresponding non-cancerous breast tissues [27], and similar results were obtained in leukocytes from venous blood of breast cancer patients compared with healthy controls [29]. These interesting observations prompt us to propose that oxidative DNA damage biomarker 8-oxodG may be utilized as a potential biomarker for cancer risk estimation, early detection, treatment and prognosis.

Nevertheless, the risk of artifactual oxidation during DNA extraction and subsequent digestion [31] is a serious problem. Moreover, the invasiveness of sample collection also has restricted its application in large-scale human studies. Urine, compared with other biofluids such as serum and saliva, has been considered to be a preferred biological matrix in clinical practice since it is easily accessible in large volumes and noninvasive to patients [32–37]. Hence, detection of 8-oxodG in urine could be the first choice for cancer risk estimation, early detection, treatment and prognosis.

In the present study, we developed a validated method for determination of 8-oxodG in human urine by ultra performance liquid chromatography-electrospray ionization tandem mass spectrometry (UPLC-ESI-MS/MS) combined with a solid-phase extraction (SPE) procedure. By the developed approach, we quantified 8-oxodG in urine samples from 60 patients with early-stage breast cancer (stage I, II), 51 patients with benign breast diseases and 73 healthy volunteers. Furthermore, logistic regression analysis and receiver operator characteristic (ROC) curve analysis were also performed to evaluate the potential of urinary 8-oxodG for distinguishing the early-stage breast cancer from benign lesions, and serving as a biomarker for risk estimation, early screening and for further detection of breast cancer.

## RESULTS

### Assay design

**Figure 1:**
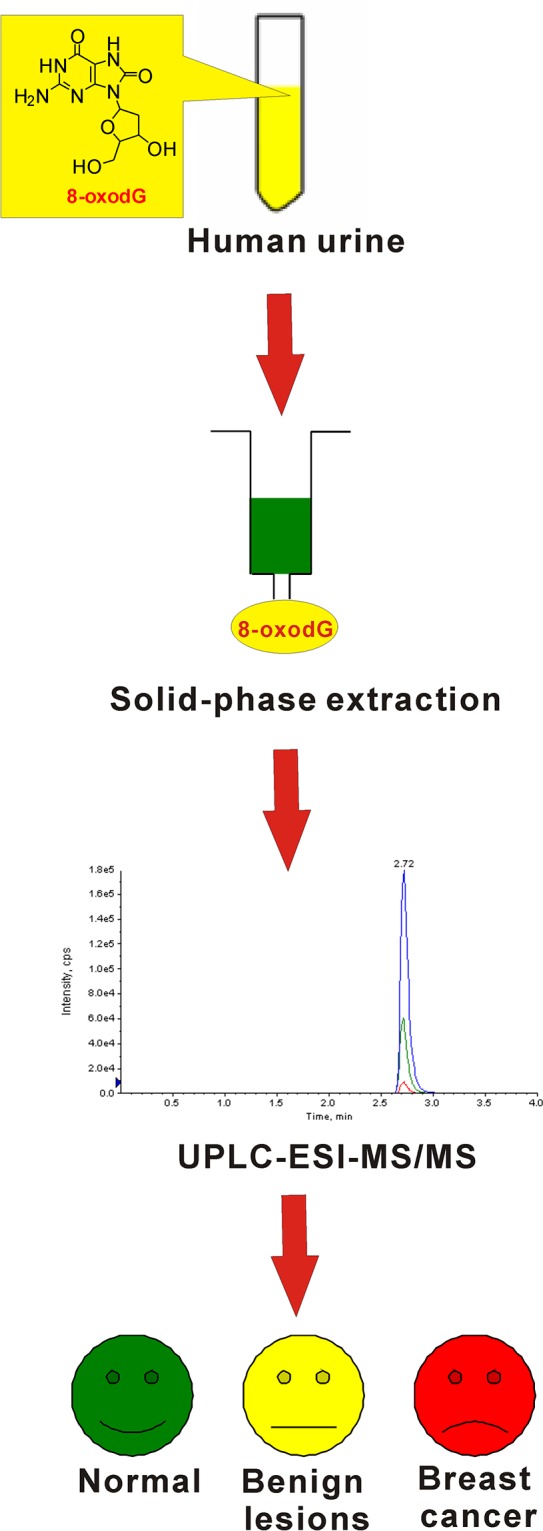
Schematic diagram of the workflow involved in detection of 8-oxodG in urine samples

There is a clinical need for discriminating early stage breast cancer from benign lesions. We therefore set out to design a reproducible and sensitive assay with high throughput to detect 8-oxodG in urine as a paradigm study. The UPLC-ESI-MS/MS based method is outlined in Figure 1. As the typical oxidative damage product of DNA, 8-oxodG excreted in urine was enriched using solid-phase extraction method. Then, pretreated urine samples from patients with early-stage breast cancer, patients with benign breast diseases and healthy volunteers were analyzed by UPLC-ESI-MS/MS to quantify the amount of 8-oxodG. Plainly a critical aspect of this MS-based assay relies on the performance of the UPLC-ESI-MS/MS method.

### Performance of the UPLC-ESI-MS/MS method

The linearity range, limit of detection, limit of quantification, precision, accuracy, recovery and matrix effect of the method were validated as the follow. In the concentration range from 1.0 to 300.0 nM, excellent linearity was obtained (*y* = 0.0287*x* + 0.032) with the correlation coefficient of R^2^ = 0.9997. The limit of detection and limit of quantification was 1 fmol and 3 fmol, respectively, which indicated that the sensitivity of the analytical method was excellent. The intraday and interday precision values ranged from 0.6% to 1.4% and 0.7% to 1.3%, respectively, and the accuracy values of the intraday study and the interday assay were in the range of 98.4%−102.1% (Supplementary Table 1). At three fortification levels, recoveries were in the range of 99.8−111.4%, with RSD values less than 3.0% (Supplementary Table 2). The value of matrix effect was 91.5%, indicating that the effect of matrix on the ionization efficiency of 8-oxodG was negligible. Besides, the analysis time of each run only takes four minutes, which indicated the method was suitable for large-scale human studies.

Moreover, in order to monitor whether the equipment system was still stable after hundreds of runs, a quality control sample was measured every fifteen urine samples. The parameters such as retention time, peak symmetry and accuracy were checked and the results revealed good system stability.

### Identification of 8-oxodG in human urine

**Figure 2:**
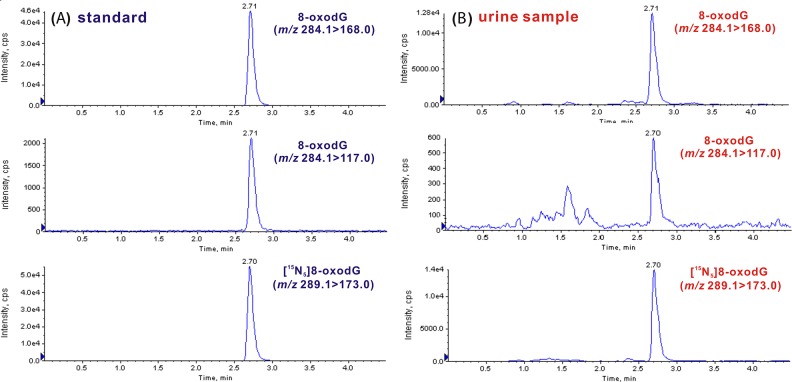
Identification of 8-oxodG in urine sample by UPLC-MS/MS **(A)** Representative chromatograms of 8-oxodG standard (*m/z* 284.1>168.0 for quantitation and *m/z* 284.1>117.0 for confirmation) and [^15^N_5_]8-oxodG internal standard (*m/z* 289.1>173.0). **(B)** Representative chromatograms of 8-oxodG and internal standard from human urine.

By the developed off-line SPE-coupled UPLC-ESI-MS/MS strategy, we further measured 8-oxodG in urine samples from 60 patients with early-stage breast cancer (stage I, II), 51 patients with benign breast disease and 73 healthy volunteers. As shown in Figure 2A, the retention time of 8-oxodG standard is 2.71 min. In urine samples (Figure 2B), the retention time of 8-oxodG is the same, which is also identical to that of the added internal standard ([^15^N_5_]8-oxodG). Moreover, 8-oxodG in urine produces an ion transition with the second highest abundance (*m/z* 284.1>117.0), which is accordant to the 8-oxodG standard. The signal ratio of the two ion transitions monitored was also evaluated by peak height and the ratio for urinary 8-oxodG is almost the same as that of the 8-oxodG standard. These results indicate that 8-oxodG in urine has the same chromatographic retention and the same tandem MS behaviors as the 8-oxodG standard, consistently confirming the presence of 8-oxodG in human urine.

### Quantification analysis of 8-oxodG in urine samples

Because of the variability among the urinary volumes and the differences in the renal glomerular function, the urinary 8-oxodG level was normalized against the corresponding creatinine level [38] and is presented as nmol of 8-oxodG/mmol of creatinine. The concentrations of all the collected urine samples were listed in Supplementary Table 3. As shown in Table 1, the measured concentration of 8-oxodG in urine samples from healthy controls is 0.26−2.67 nmol/mmol creatinine, and the average concentration is 1.12 ± 0.57 nmol/mmol creatinine (n = 73). The concentration of 8-oxodG in urine from patients with benign breast diseases is in the range of 0.20−3.46 nmol/mmol creatinine, and the average concentration is 1.09 ± 0.65 nmol/mmol creatinine (n = 51). For patients with early-stage breast cancer, the concentration of 8-oxodG in urine is in the range of 0.46−6.65 nmol/mmol creatinine, and the average concentration is 1.88 ± 1.28 nmol/mmol creatinine (n = 60).

**Table 1:** Measured concentrations of 8-oxodG in human urine samples

Group	Average concentrationxs (nmol/mmol creatinine)	Concentration range (nmol/mmol creatinine)
Normal (n = 73)	1.12 ± 0.57	0.26−2.67
Benign breast disease (n = 51)	1.09 ± 0.65	0.20−3.46
Non-cancerous (n = 124)	1.11 ± 0.60	0.20−3.46
Breast cancer (n = 60)	1.88 ± 1.28	0.46−6.65

**Figure 3:**
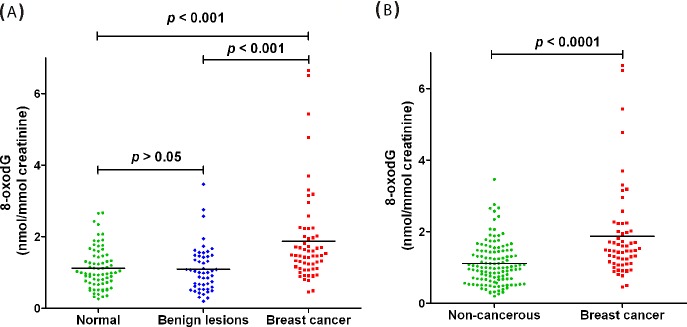
Quantification and statistical analysis of 8-oxodG in human urine samples **(A)** 8-oxodG levels in healthy control group, patients with benign breast disease group and patients with breast cancer group. **(B)** 8-oxodG levels in non-cancerous group and patients with breast cancer group.

The concentration of urinary 8-oxodG was markedly increased in patients with early-stage breast cancer compared to the healthy controls (*p* < 0.001, (Figure 3A). It is interesting to find that the content of urinary 8-oxodG in patients with early-stage breast cancer was also dramatically increased compared to that in patients with benign breast diseases (*p* < 0.001). However, there was no significant difference of urinary 8-oxodG level between benign breast diseases group and healthy control group (*p* > 0.05). And thus benign breast diseases group and healthy control group were integrated as a non-cancerous group. The concentration of 8-oxodG in urine from non-cancerous group is in the range of 0.20−3.46 nmol/mmol creatinine, and the average concentration is 1.11 ± 0.60 nmol/mmol creatinine (n = 124). Significant difference was observed between non-cancerous group and breast cancer group (Figure 3B). These results indicate that the oxidative damage was much more serious in patients with breast cancer than patients with benign breast diseases or healthy controls, and the content of 8-oxodG in urine could be utilized for discriminating early-stage breast cancer from benign breast diseases, and served as an indicator of breast cancer.

### Logistic regression analysis reveals the potential of 8-oxodG as a biomarker of breast cancer

In order to further evaluate the correlation of the development of breast cancer with respect to urinary 8-oxodG concentration and age, logistic regression analysis was carried out. The values of 8-oxodG was used as continuous variable and age was adjusted as a categorical variable. Considering the mean age of the patients and healthy controls, sever thresholds of age including 45, 50, 55 and 60 years old were tested and we found that a best fitting effect of the logistic regression model was obtained when the threshold was set as 55 years old. The results demonstrated that individuals with higher urinary 8-oxodG level and older age were more possibly to develop breast cancer (Table 2).

**Table 2:** Logistic regression analysis of factors associated with breast cancer

Variable		Odds ratio	95% CI	*p*
Urinary 8-oxodG concentration		2.605	1.549–4.381	< 0.001
Age	≥ 55	3.442	1.571–7.540	< 0.005
	< 55	1	–	–

Besides, a logistic regression model consisted of urinary 8-oxodG concentration and age was established to predict breast cancer risk, and the probability was calculated according to the formula shown below.

$$\text{Probability }=\frac{\exp(-2.378\;+\;0.957\;\times \text{ urinary 8-oxodG }+\;1.236\;\times \text{ grade of age})}{1+\exp(-2.378\;+\;0.957\;\times \text{ urinary 8 -oxodG }+\;1.236\;\times \text{ grade of age})}$$

**Figure 4:**
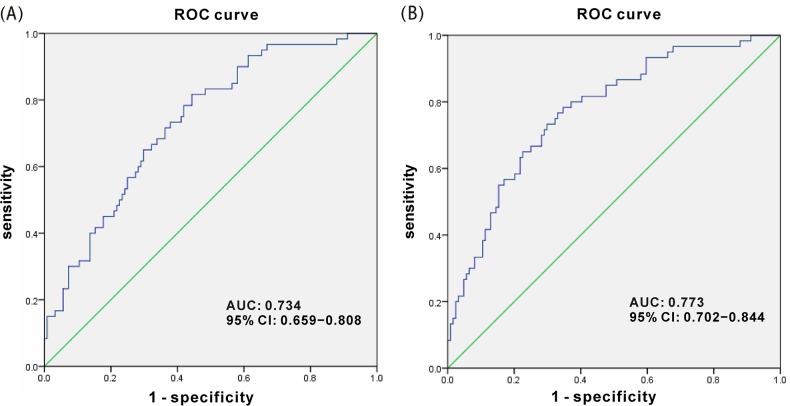
ROC curve for urinary 8-oxodG score **(A)** Logistic regression model only concerning urinary 8-oxodG concentration. **(B)** Logistic regression model concerning urinary 8-oxodG concentration and age.

The value of grade of age is set at 1 or 0 when the age is higher or lower than 55 years old, respectively. The cutoff value of this model was 0.5, which meant that individuals with a probability greater than 0.5 had significantly higher risk of developing breast cancer than those with a probability less than 0.5. In addition, a logistic regression model only concerning urinary 8-oxodG concentration was established and ROC analysis was performed. As illustrated in Figure 4A, the area under the curve (AUC) is 0.734, 95% CI 0.659−0.808, *p* < 0.0001. And the sensitivity and specificity of urinary 8-oxodG alone for the diagnosis of breast cancer is 0.733 and 0.621, respectively. On the other hand, for the logistic regression model consisted of urinary 8-oxodG concentration and age, a higher AUC value of the ROC curve was obtained, which implies good fitting effect of this logistic regression model and more effective detection. Moreover, the sensitivity and specificity of urinary 8-oxodG combined with age is 0.767 and 0.669, respectively, which are both higher. As shown in Figure 4B, urinary 8-oxodG was highly effective in the detection of breast cancer with AUC being 0.773, 95% CI 0.702−0.844, *p* < 0.0001.

## DISCUSSION

Nowadays, breast cancer becomes more and more common and highly fatal in women. However, unlike the cases of prostate specific antigen (PSA) for prostatic cancer and α-fetoprotein (AFP) for liver cancer, up to now, there was no biomarker available for early warning of breast cancer in clinical practice except that invasive genetic test of BRCA1/2 mutation was recommended to evaluate the risk of hereditary breast cancer [39]. Although mammography is currently utilized for breast cancer screening, the high false positive rate usually results in further examination including expensive magnetic resonance imaging and invasive biopsies. Thus, a non-invasive detection technique, as a companion tool together with mammography, for the screening of breast cancer, particularly for discrimination early cancer from benign lesions, is urgently needed in clinical practice.

8-oxodG, as a representative product of oxidative damage to DNA, plays a significant role in carcinogenesis and other disease processes [40]. In the past years, great efforts have been devoted to the study of 8-oxodG in DNA isolated from cell lines or tissue samples [27–30]. However, the invasiveness of sample collection and the artifactual oxidation during DNA extraction and digestion are the main issues which restrict its application in clinic practice. Recently, biomarker discovery from urine samples has attracted increasing attentions [32–37]. In previous studies, 8-oxodG in urine was analyzed by high-performance liquid chromatography with electrochemical detection (HPLC-ECD) or enzyme-linked immunosorbent assay (ELISA) to assess the association between 8-oxodG excretion and breast cancer risk [41, 42]. However, HPLC-ECD requires multi-dimensional chromatography and thorough sample cleanup to reduce the risk of overlapping peaks, and ELISA frequently overestimates urinary 8-oxodG levels due to the lack of specificity. Urinary 8-oxodG was also evaluated in breast cancer patients before and after tumor removal by LC-MS/MS [43], and 8-oxodG level was found to be significantly higher in pre-operative patients than in post-operative patients, but patients with benign breast diseases were not involved. Hence, determination of 8-oxodG in urine by UPLC-ESI-MS/MS is desirable to provide some insights for early detection of breast cancer, particularly for distinguishing early-stage breast cancer from benign lesions.

In this study, we utilized solid-phase extraction coupled with UPLC-ESI-MS/MS to analyze 184 urine samples. The use of mass spectrometer under multiple reaction monitoring (MRM) mode can provide high selectivity. In addition, solid-phase extraction (SPE) applied for the cleanup and enrichment of the target metabolites was usually combined with LC-ESI-MS/MS to avoid possible ionization source contamination and ion suppression caused by coexisting ingredients in urine. Moreover, the utilization of stable isotope-labeled standards can compensate for the loss of analyte during sample preparation.

Many studies which aim at revealing novel cancer biomarkers just compared healthy control group with breast cancer patient group. However, it is important to introduce benign breast diseases group for discovery of cancer biomarker since it could eliminate the compounds with similarities between the two patient groups. We demonstrated that the concentration of urinary 8-oxodG in patients with early-stage breast cancer was significantly higher than that in healthy controls. Moreover, it is interesting to find that the concentration of urinary 8-oxodG in patients with early-stage breast cancer was also markedly increased relative to that in patients with benign breast diseases, whereas no significant difference of urinary 8-oxodG level was observed between benign breast diseases group and healthy control group. In addition, there was significant difference between early-stage breast cancer group and non-cancerous group which consisted of benign breast diseases patients and healthy controls.

Logistic regression analysis and receiver operator characteristic (ROC) curve analysis were also carried out, and the results revealed that urinary 8-oxodG could be used in prediction of breast cancer risk and highly effective detection of breast cancer. All of these findings suggest that oxidative stress plays important roles in the development of breast cancer and the marked increase of oxidative DNA damage biomarker 8-oxodG in urine may serve as a potential non-invasive biomarker for the risk estimation, early warning and detection of breast cancer, particularly for distinguishing early-stage breast cancer from benign lesions.

## MATERIALS AND METHODS

### Chemicals

Chromatographic grade methanol (MeOH) used for HPLC was purchased from Merck (Darmstadt, Germany). 8-oxo-7,8-dihydro-2′-deoxyguanosine (8-oxodG), acetic acid (CH_3_COOH), were purchased from Sigma-Aldrich (St Louis, MO, USA). Isotopically labeled internal standard (IS), [^15^N_5_]8-oxo-7,8-dihydro-2′-deoxyguanosine ([^15^N_5_]8-oxodG) was purchased from Cambridge Isotope Laboratories Inc. (Andover, MA, USA). Water used throughout all experiments was purified using a Milli-Q water purification apparatus (Millipore, Milford, MA, USA). 8-oxodG and [^15^N_5_]8-oxodG were dissolved in water to a stock concentration of 1 mM and 0.1 mM, respectively. Aliquots were stored at −80°C until use. When required, these were diluted to 1 μM in water.

### Sample collection

This study was approved by the Institutional Review Board of Medical Research, The Second Affiliated Hospital, Zhejiang University School of Medicine (SAHZU) and all experiments were carried out in accordance with the approved guidelines. The patients with breast cancer or benign lesion were pathologic confirmed and had not been treated with surgical operation, radiotherapy nor chemotherapy. Besides, these patients were excluded from any other types of cancer. The patients with breast cancer recruited were at the early stages (stage I, II) according to the clinical staging. Benign breast lesions included fibroadenomas, hyperplasia and cysts. These female individuals were recruited from the Department of Surgical Oncology at SAHZU. Healthy women were recruited from healthy examination volunteers. All subjects were excluded from diabetes, cardiovascular diseases and other types of cancer, and gave written informed consent prior to participation.

A total of 60 patients with breast cancer (mean age of 53.6 ± 11.4 years, range 32–89 years) with TNM stage I (n = 34) and stage II (n = 26), 51 patients with benign breast diseases including breast fibroadenoma, galactoma, cystic hyperplasia and intraductal papilloma (mean age of 45.8 ± 10.7 years, range 26–77 years), and 73 healthy volunteers (mean age of 43.7 ± 9.2 years, range 21–64 years) were recruited. All subjects were asked to provide mid-stream early-morning urine specimens. After urine collection the samples were frozen immediately and stored at −80°C in the dark until analysis.

### Solid-phase extraction

The urine samples were fully thawed on the day of extraction at room temperature and centrifuged at 13000 rpm for 15 min at 4°C. 500 μL of supernatant was mixed with 500 μL of water, and then spiked with 10 pmol of [^15^N_5_]8-oxodG internal standard (IS). The samples were pretreated using Oasis HLB (3.0 mL, 60 mg) cartridges (Waters, Milford, MA, USA). Each cartridge was activated with 1.0 mL of methanol and then equilibrated with 1.0 mL of water. Subsequently, urine samples were loaded. The cartridges were washed with 1.0 mL of methanol/water of 5:95 (v/v), followed by the final elution using 1.0 mL of methanol/water of 1:1 (v/v). The eluted fractions were evaporated under vacuum. The dried urine samples were reconstituted in 200 μL of water for UPLC-ESI-MS/MS analysis. In addition, aliquots of urine supernatant were also assayed for creatinine to provide a correction factor for urine concentration because the urinary excretion rate of creatinine is relatively constant over time (Department of Laboratory Medicine, SAHZU).

### UPLC-ESI-MS/MS analysis

The UPLC analysis was performed on an Acquity UPLC system (Waters, Milford, MA, USA), equipped with a binary solvent manager, an autosampler and a column heater. The column was an Acquity UPLC BEH C18 (2.1 mm × 100 mm, 1.7 μm particle size, Waters) and the temperature was maintained at 40°C. The mobile phase was (A) 0.1% acetic acid, and (B) methanol. An isocratic mode using 92.5% A and 7.5% B was used to achieve the desired sample separation with a flow rate of 0.25 mL/min. Samples were maintained at 4°C throughout. Each sample was analyzed at least three times and an injection volume of 5 μL was used. The flow from the column was directed to the mass spectrometer.

The MS detection was performed on a 4000 QTRAP mass spectrometer (AB SCIEX, Foster City, CA, USA) equipped with an ESI ion source (Turbospray) operated in positive ion mode. Instrument control, data acquisition, and processing were performed using the associate Analyst 1.5.2 software. MS parameters including collision energy were optimized by direct continuous pump infusion of standard solutions of the 8-oxodG and [^15^N_5_]8-oxodG (10 μM) individually at a flow rate of 10 μL/min in the mass spectrometer. Collision-induced dissociation (CID) experiments [44] of 8-oxodG and [^15^N_5_]8-oxodG were performed in product ion scan mode and the spectra were illustrated in Supplementary Figure 1. For 8-oxodG, two transitions between precursor ion and the two most abundant fragment ions were monitored: the transition *m/z* 284.1>168.0 for quantitative determination and the transition *m/z* 284.1>117.0 for qualitative analysis. And for the isotopically labeled internal standard, the transition *m/z* 289.1>173.0 was monitored for quantitative determination. Multiple reaction monitoring (MRM) was carried out using the instrumental parameters summarized in Supplementary Table 4. To increase sensitivity, the ion source temperature (TEM) was set at 550°C, and the ion spray voltage was set at 5.5 kV. Ion source gas 1 (GS1) and ion source gas 2 (GS2) used as the nebulizing and drying gases were set at 60 and 40 psi, respectively. Curtain gas (CUR) was set at 35 psi.

### Statistical analysis

All the statistical analyses were performed using SPSS statistics 20.0 software (IBM, Armonk, NY, USA). Oneway ANOVA was applied to compare concentrations of urinary 8-oxodG in breast cancer group, benign breast diseases group and healthy controls. Mann-Whitney*U* test was applied to evaluate the differences of concentration levels of urinary 8-oxodG between patients with breast cancer and non-cancerous group (including benign breast diseases group and normal volunteers). Logistic regression analysis was used to determine the relationship between breast cancer detection and other factors, such as age and urinary 8-oxodG concentration. Besides, a logistic regression model was established to predict breast cancer risk. Receiver operator characteristic (ROC) curve analysis was applied to evaluate the fitting effect of logistic regression model. Statistical tests were two sided and *p* < 0.05 was considered statistically significant.

## SUPPLEMENTARY MATERIALS FIGURE AND TABLES




