# Supplementary material for: Discriminating patients with early-stage breast cancer from benign lesions by detection of oxidative DNA damage biomarker in urine
Source: Oncotarget. 2017 May 12;8(32):53100–9. doi: 10.18632/oncotarget.17831 (PMC5581095; doi:10.18632/oncotarget.17831)
Supplement: Supplementary file 1 [file oncotarget-08-53100-s001.pdf]

Discriminating patients with early-stage breast cancer from benign lesions by detection of oxidative DNA damage biomarker in urine

SUPPLEMENTARY FIGURE AND TABLES

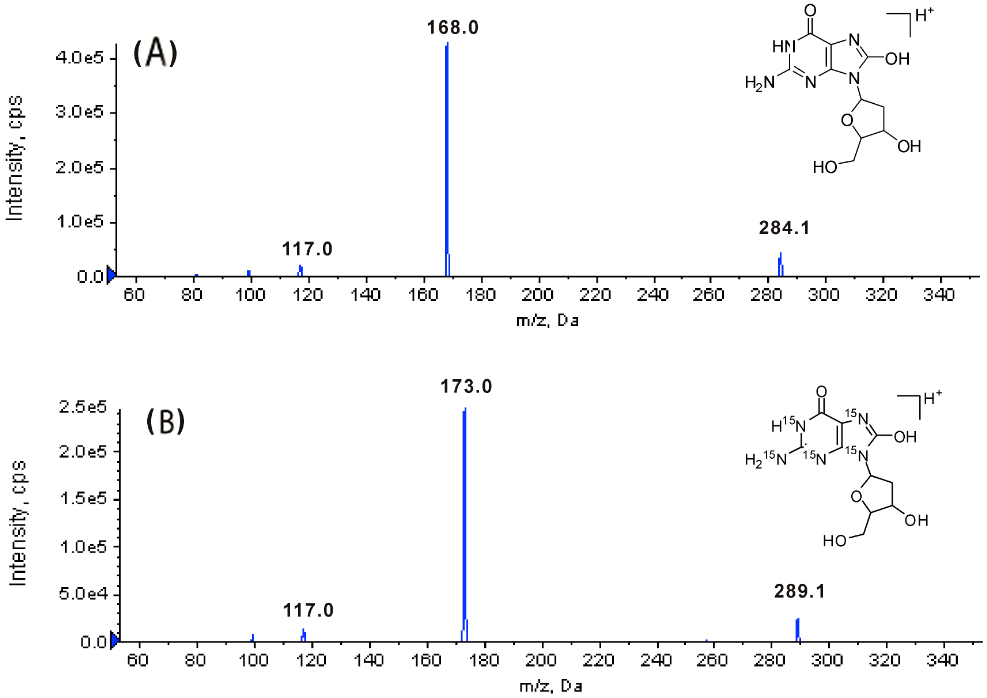

Supplementary Figure 1: The collision-induced dissociation (CID) mass spectra of (A) 8-oxodG and (B) [$^{15}N_5$]8-oxodG.

Supplementary Table 1: Precision and accuracy for 8-oxodG QC samples at three different concentrations

	Levels of 8-oxodG		
	LQC (10 nM)	MQC (50 nM)	HQC (200 nM)
Intraday (n = 9)			
Mean ± SD (nM)	9.84 ± 0.13	50.78 ± 0.50	199.29 ± 1.26
RSD (%)	1.4	1.0	0.6
Accuracy (%)	98.4	101.6	99.6
Interday (n = 3)			
Mean ± SD (nM)	9.86 ± 0.11	51.05 ± 0.64	200.05 ± 1.48
RSD (%)	1.1	1.3	0.7
Accuracy (%)	98.6	102.1	100.0

Supplementary Table 2: Recoveries of the developed off-line SPE-coupled UPLC-ESI-MS/MS method obtained at three different spiking levels

	Added amount of 8-oxodG (nM)			
	**0**	**5 (low)**	**20 (medium)**	**60 (high)**
Mean ± SD (nM)	12.27 ± 0.13	17.84 ± 0.51	32.64 ± 0.92	72.17 ± 1.27
Average recovery (%)	–	111.4	101.9	99.8
RSD (%)	–	2.8	2.8	1.8

Supplementary Table 3: General information and urinary 8-oxodG concentration of healthy volunteers, patients with benign breast disease and patients with breast cancer

See Supplementary File 1

Supplementary Table 4: The optimized MS conditions used for the analysis of 8-oxodG

**Compound**	**MRM ion transition (*m/z*)**	**DP (V)**	**CE (V)**	**EP (V)**	**CXP (V)**	**Dwell time (ms)**
8-oxodG	284.1>168.0	45.0	18.0	10.0	13.0	150
	284.1>117.0	45.0	22.0	10.0	5.0	150
[$^{15}N_5$]8-oxodG	289.1>173.0	45.0	18.0	10.0	13.0	150

DP: declustering potential; CE: collision energy; EP: entrance potential; CXP: collision cell exit potential
