# Supplementary material for: Discriminating patients with early-stage breast cancer from benign lesions by detection of oxidative DNA damage biomarker in urine
Source: Oncotarget. 2017 May 12;8(32):53100–9. doi: 10.18632/oncotarget.17831 (PMC5581095; doi:10.18632/oncotarget.17831)
Supplement: Supplementary file 2 [file oncotarget-08-53100-s002.docx]

**Supplementary Table 3: General information and urinary 8-oxodG concentration of healthy volunteers, patients with benign breast disease and patients with breast cancer**

**No.**

**Age (years)**

**Sex**

**TNM Stage**

**Urinary 8-oxodG concentration**

**(nmol/mmol creatinine)**

Normal-1

27

F

-

0.804±0.005

Normal-2

27

F

-

1.106±0.041

Normal-3

51

F

-

1.466±0.090

Normal-4

55

F

-

1.245±0.129

Normal-5

36

F

-

1.627±0.027

Normal-6

51

F

-

0.665±0.010

Normal-7

30

F

-

0.709±0.015

Normal-8

29

F

-

0.570±0.073

Normal-9

46

F

-

1.493±0.048

Normal-10

50

F

-

0.963±0.004

Normal-11

50

F

-

0.265±0.014

Normal-12

47

F

-

0.939±0.091

Normal-13

52

F

-

0.983±0.110

Normal-14

53

F

-

2.654±0.015

Normal-15

62

F

-

0.960±0.055

Normal-16

41

F

-

0.725±0.018

Normal-17

44

F

-

0.998±0.030

Normal-18

51

F

-

0.513±0.054

Normal-19

35

F

-

0.853±0.021

Normal-20

39

F

-

0.907±0.041

Normal-21

47

F

-

0.505±0.005

Normal-22

41

F

-

1.430±0.087

Normal-23

50

F

-

0.933±0.082

Normal-24

41

F

-

1.150±0.024

Normal-25

55

F

-

1.116±0.018

Normal-26

43

F

-

1.139±0.014

Normal-27

48

F

-

0.563±0.011

Normal-28

35

F

-

1.594±0.061

Normal-29

52

F

-

1.203±0.026

Normal-30

55

F

-

1.672±0.075

Normal-31

46

F

-

0.539±0.017

Normal-32

42

F

-

0.490±0.049

Normal-33

50

F

-

1.237±0.029

Normal-34

45

F

-

1.776±0.058

Normal-35

64

F

-

1.760±0.043

Normal-36

48

F

-

2.666±0.024

Normal-37

41

F

-

1.257±0.004

Normal-38

60

F

-

2.075±0.103

Normal-39

38

F

-

1.032±0.037

Normal-40

42

F

-

1.332±0.050

Normal-41

57

F

-

0.818±0.036

Normal-42

47

F

-

2.062±0.031

Normal-43

36

F

-

0.894±0.097

Normal-44

41

F

-

0.351±0.010

Normal-45

51

F

-

1.273±0.009

Normal-46

44

F

-

0.510±0.022

Normal-47

51

F

-

2.431±0.050

Normal-48

39

F

-

0.322±0.009

Normal-49

45

F

-

0.513±0.059

Normal-50

48

F

-

1.312±0.024

Normal-51

50

F

-

1.881±0.155

Normal-52

26

F

-

0.893±0.012

Normal-53

47

F

-

0.688±0.009

Normal-54

26

F

-

0.760±0.029

Normal-55

32

F

-

1.646±0.026

Normal-56

23

F

-

1.123±0.027

Normal-57

49

F

-

1.907±0.005

Normal-58

21

F

-

1.044±0.031

Normal-59

40

F

-

0.971±0.016

Normal-60

48

F

-

0.392±0.012

Normal-61

48

F

-

1.467±0.026

Normal-62

41

F

-

0.322±0.051

Normal-63

45

F

-

0.949±0.027

Normal-64

37

F

-

0.994±0.051

Normal-65

51

F

-

1.312±0.153

Normal-66

31

F

-

0.673±0.012

Normal-67

37

F

-

0.794±0.009

Normal-68

49

F

-

1.891±0.052

Normal-69

30

F

-

1.740±0.024

Normal-70

45

F

-

0.410±0.101

Normal-71

40

F

-

0.768±0.049

Normal-72

43

F

-

0.512±0.014

Normal-73

51

F

-

2.345±0.234

Benign-1

77

F

-

2.573±0.072

Benign-2

34

F

-

2.759±0.148

Benign-3

45

F

-

1.666±0.041

Benign-4

45

F

-

0.912±0.026

Benign-5

52

F

-

0.985±0.022

Benign-6

38

F

-

1.623±0.043

Benign-7

51

F

-

0.474±0.008

Benign-8

43

F

-

1.039±0.029

Benign-9

43

F

-

0.198±0.008

Benign-10

32

F

-

0.623±0.015

Benign-11

26

F

-

0.428±0.011

Benign-12

47

F

-

1.113±0.008

Benign-13

58

F

-

1.484±0.010

Benign-14

42

F

-

1.590±0.059

Benign-15

37

F

-

0.464±0.014

Benign-16

52

F

-

1.354±0.063

Benign-17

38

F

-

0.532±0.008

Benign-18

34

F

-

0.506±0.005

Benign-19

46

F

-

0.307±0.005

Benign-20

50

F

-

0.391±0.011

Benign-21

55

F

-

1.553±0.071

Benign-22

57

F

-

1.737±0.055

Benign-23

69

F

-

1.053±0.058

Benign-24

60

F

-

0.662±0.043

Benign-25

39

F

-

0.289±0.005

Benign-26

55

F

-

1.459±0.047

Benign-27

32

F

-

0.997±0.009

Benign-28

45

F

-

0.745±0.026

Benign-29

49

F

-

0.491±0.007

Benign-30

42

F

-

0.534±0.041

Benign-31

44

F

-

1.087±0.035

Benign-32

42

F

-

1.072±0.004

Benign-33

29

F

-

0.672±0.001

Benign-34

45

F

-

0.525±0.020

Benign-35

38

F

-

0.997±0.059

Benign-36

37

F

-

1.429±0.008

Benign-37

51

F

-

0.884±0.017

Benign-38

33

F

-

1.262±0.029

Benign-39

31

F

-

0.847±0.059

Benign-40

36

F

-

0.691±0.016

Benign-41

53

F

-

1.446±0.021

Benign-42

60

F

-

1.947±0.049

Benign-43

51

F

-

1.314±0.023

Benign-44

38

F

-

1.084±0.027

Benign-45

46

F

-

0.592±0.026

Benign-46

46

F

-

1.483±0.033

Benign-47

52

F

-

0.683±0.025

Benign-48

50

F

-

1.301±0.022

Benign-49

54

F

-

0.850±0.020

Benign-50

35

F

-

1.560±0.025

Benign-51

70

F

-

3.461±0.031

Cancer-1

52

F

I

1.029±0.007

Cancer-2

55

F

II

1.463±0.035

Cancer-3

48

F

I

1.484±0.047

Cancer-4

44

F

II

1.703±0.016

Cancer-5

65

F

I

1.732±0.017

Cancer-6

55

F

II

1.335±0.002

Cancer-7

51

F

II

0.778±0.017

Cancer-8

58

F

II

1.538±0.024

Cancer-9

59

F

I

1.116±0.015

Cancer-10

79

F

I

1.948±0.060

Cancer-11

89

F

II

1.686±0.052

Cancer-12

43

F

I

0.495±0.007

Cancer-13

47

F

II

0.916±0.038

Cancer-14

48

F

I

2.027±0.046

Cancer-15

68

F

I

1.327±0.039

Cancer-16

47

F

II

1.488±0.040

Cancer-17

50

F

II

1.243±0.019

Cancer-18

40

F

II

0.998±0.044

Cancer-19

42

F

I

0.943±0.010

Cancer-20

32

F

I

1.478±0.027

Cancer-21

49

F

I

0.926±0.014

Cancer-22

53

F

I

1.720±0.014

Cancer-23

75

F

II

1.611±0.008

Cancer-24

66

F

II

0.893±0.009

Cancer-25

40

F

I

2.230±0.030

Cancer-26

54

F

I

1.652±0.037

Cancer-27

47

F

I

2.236±0.036

Cancer-28

58

F

I

0.891±0.012

Cancer-29

65

F

I

1.112±0.020

Cancer-30

76

F

I

1.263±0.038

Cancer-31

52

F

II

1.467±0.010

Cancer-32

39

F

I

6.651±0.088

Cancer-33

55

F

I

1.106±0.027

Cancer-34

49

F

I

2.266±0.111

Cancer-35

49

F

I

6.506±0.217

Cancer-36

67

F

II

5.433±0.188

Cancer-37

56

F

I

3.188±0.043

Cancer-38

56

F

I

2.577±0.036

Cancer-39

59

F

I

2.013±0.067

Cancer-40

63

F

I

3.699±0.573

Cancer-41

49

F

II

1.078±0.081

Cancer-42

40

F

II

1.608±0.211

Cancer-43

52

F

II

0.912±0.008

Cancer-44

69

F

II

2.245±0.021

Cancer-45

43

F

I

0.463±0.002

Cancer-46

46

F

II

1.081±0.008

Cancer-47

60

F

II

1.164±0.016

Cancer-48

38

F

I

4.776±0.190

Cancer-49

45

F

I

3.148±0.088

Cancer-50

41

F

II

1.316±0.128

Cancer-51

60

F

II

3.310±0.159

Cancer-52

39

F

I

1.424±0.026

Cancer-53

51

F

II

1.430±0.024

Cancer-54

47

F

II

1.301±0.032

Cancer-55

61

F

I

1.978±0.026

Cancer-56

41

F

I

1.693±0.037

Cancer-57

65

F

I

1.237±0.007

Cancer-58

72

F

II

1.462±0.010

Cancer-59

45

F

I

1.816±0.006

Cancer-60

53

F

II

2.963±0.004
